# Recording, labeling, and transfection of single neurons in deep brain structures

**DOI:** 10.14814/phy2.12246

**Published:** 2015-01-19

**Authors:** Bowen Dempsey, Anita J. Turner, Sheng Le, Qi‐Jian Sun, Lama Bou Farah, Andrew M. Allen, Ann K. Goodchild, Simon McMullan

**Affiliations:** Australian School of Advanced Medicine, Macquarie University, Sydney, 2109, NSW, Australia; Department of Physiology, The University of Melbourne, Parkville, 3010, VIC, Australia

**Keywords:** Electroporation, gene delivery, juxtacellular, labeling

## Abstract

Genetic tools that permit functional or connectomic analysis of neuronal circuits are rapidly transforming neuroscience. The key to deployment of such tools is selective transfection of target neurons, but to date this has largely been achieved using transgenic animals or viral vectors that transduce subpopulations of cells chosen according to anatomical rather than functional criteria. Here, we combine single‐cell transfection with conventional electrophysiological recording techniques, resulting in three novel protocols that can be used for reliable delivery of conventional dyes or genetic material in vitro and in vivo. We report that techniques based on single cell electroporation yield reproducible transfection in vitro*,* and offer a simple, rapid and reliable alternative to established dye‐labeling techniques in vivo, but are incompatible with targeted transfection in deep brain structures. In contrast, we show that intracellular electrophoresis of plasmid DNA transfects brainstem neurons recorded up to 9 mm deep in the anesthetized rat. The protocols presented here require minimal, if any, modification to recording hardware, take seconds to deploy, and yield high recovery rates in vitro (dye labeling: 89%, plasmid transfection: 49%) and in vivo (dye labeling: 66%, plasmid transfection: 27%). They offer improved simplicity compared to the juxtacellular labeling technique and for the first time offer genetic manipulation of functionally characterized neurons in previously inaccessible brain regions.

## Abstract (precis)

The ability to label individual neurons after electrophysiological characterization of their functional properties is a foundational technique in neuroscience. A number of approaches that achieve this goal have been described, but all are technically challenging. Here, we describe a simple approach that is rapid, reliable, and compatible with delivery of conventional dyes or large plasmid DNA molecules.

## Introduction

Techniques that combine electrophysiological recording of neuronal activity with dye labeling have been used to address fundamental questions about the relationship between neurochemistry, morphology, and cell behavior (Schreihofer and Guyenet 1997; Bevan 1998; Mileykovskiy et al. 2005; Noseda et al. 2010; Jiang et al. 2013). Historically, investigators have used three main strategies to introduce dye from a recording pipette to the cell interior. In the first, intracellular access is obtained by impalement of the neuron with a sharp electrode and fluorescent dyes or biotin conjugates are deposited by intracellular electrophoresis (Stretton and Kravitz 1968; Horikawa and Armstrong 1988). In the second, whole cell access is obtained using a low‐resistance patch pipette and dye is passively dialyzed into the cell (Edwards et al. 1989; Pickering et al. 1991). In the third approach, an extracellular recording electrode is positioned in close contact with the cell membrane (a “juxtacellular” position) and a train of 200 ms long positive current pulses up to 10 nA in amplitude is used to initiate and maintain membrane electroporation and simultaneously eject positively charged dyes, typically over a period of 2–30 min (Pinault 1996; for review, see Pinault 2011).

All three approaches are technically difficult and require experience and skill for efficient use, particularly in vivo. The quality of labeling obtained using the juxtacellular approach is generally inferior to that obtained using intracellular dye deposition; however, the technical difficulty associated with maintaining stable sharp recordings or obtaining whole cell access in deep brain regions in vivo has led to the ascendency of Pinault's juxtacellular technique as the gold‐standard approach for labeling functionally identified neurons.

Recent advances in molecular biology have provided incentives for the development of single‐cell labeling techniques that are compatible with intracellular nucleotide delivery. The major challenge associated with delivery of genetic material is the large molecular weight of gene constructs and the high copy number required for efficient transfection. For example, the molecular weight of the plasmid that encodes yellow fluorescent protein, pCAG‐YFP (MW 1.8 MDa) is approximately 6000 times greater than that of neurobiotin (MW 286 Da). This obstacle has been overcome using two approaches. First, as with traditional dyes, plasmids can be dialyzed into neurons during low‐resistance whole cell recordings (Rancz et al. 2011). Although the transfection rate associated with this approach is high (56%: Rancz et al. 2011) and the use of whole‐cell patch recordings in vivo is becoming more commonplace, this approach is still restricted to more superficial brain regions as whole‐cell recordings become difficult to obtain beyond about 2 mm deep (Margrie et al. 2002; Schramm et al. 2014).

An alternative approach combines conventional electrophysiological recording methods with single cell electroporation (SCE) (Haas et al. 2001; Rae and Levis 2002; Rathenberg et al. 2003; Bestman et al. 2006; Steinmeyer and Yanik 2012). In common with the juxtacellular technique, SCE uses voltage trains to induce localized dielectric breakdown of the cell membrane and drive charged molecules from the pipette into the cell, but differs in terms of the duration (~1 ms), frequency (50–1000 Hz), and amplitude of pulses (~10 V, equivalent to ~500 nA assuming a series resistance of 20 MΩ). SCE is an efficient and quick transfection method, but suffers some limitations: first, it is critically dependent on gentle contact between the pipette and target cell, meaning its use is largely restricted to preparations in which direct visualization of the cell is possible (Rathenberg et al. 2003; Kitamura et al. 2008; Judkewitz et al. 2009). Furthermore, the voltages required for efficient SCE are beyond the limits of commercially available voltage‐clamp amplifiers, meaning SCE cannot readily be combined with electrophysiological characterization of target neurons.

Three recent reports detail amplifier modifications and protocols that combine traditional electrophysiological recordings with SCE, allowing transfection of recorded neurons in vitro (Daniel et al. 2013) or, within superficial layers of the cortex, in vivo (Cohen et al. 2013; Oyama et al. 2013). These achievements represent an important technical landmark that, in common with the whole‐cell transfection technique (Rancz et al. 2011), may prove valuable to investigators studying neurons in easily accessible brain regions. However, their applicability to neurons in deep or fibrous regions of the adult brain remains unproven.

Our group has a long‐standing interest in the anatomy, behavior, and network dynamics of autonomic and respiratory nuclei deep in the ventrolateral medulla of the rat (McMullan et al. 2008; Sevigny et al. 2008; Burke et al. 2011). These neurons are located up to 9 mm deep to the cerebellar surface, lie intermingled with large fiber tracts, and are not amenable to whole‐cell recordings in recovery experiments. The objective of the current study was to develop a technique that can be used for the targeted transfection of electrophysiologically profiled neurons in deep brain regions. We first independently developed an approach that combines extracellular recording of unit activity with SCE. We then validated its efficacy in vitro and extensively tested its suitability for transfection of neurons recorded >2 mm deep in the brainstem. We report that SCE‐based approaches provide good transfection efficiency in vitro and can be used in vivo for dye‐labeling as a simple and reliable alternative to the juxtacellular technique. However, in our hands SCE did not result in reliable transfection in vivo. To circumvent this limitation we describe a protocol for intracellular electrophoresis of DNA and show that this is a more useful approach.

## Methods

Ethical Approval: All experiments were approved by Macquarie University Animal Ethics Committee and conformed to the Australian Code of Practice for the Care and Use of Animals for Scientific Purposes.

### General preparation

#### Preparation of brain slices for *in vitro* electroporation

P2‐8 Sprague–Dawley rat pups of either sex were anesthetized with isoflurane and decapitated when arreflexic. The head was submerged in ice‐cold carbogen‐bubbled artificial cerebrospinal fluid (ACSF, in mm: 125 NaCl, 25 NaHCO_3_, 3 KCL, 1.25 NaH_2_PO_4_.H_2_0, 10 glucose, 2 CaCl_2_, 1 MgCl_2_). The brain was dissected and 250 *μ*m slices of hippocampus, cortex, or brainstem were cut using a vibratome in ice‐cold ACSF. Slices were maintained and recorded at 34°C in ACSF. In some cases spontaneous activity was enhanced by superfusing slices in 5–12 mm [K^+^] ACSF (Onimaru and Homma 2007).

Organotypic slice cultures of hippocampus, cortex, brainstem, and cerebellum were prepared as previously described (De Simoni and Yu 2006). Cultures were maintained on organotypic culture mesh inserts (Millipore, Billerica, MA; PICM03050) in six well dishes, submerged in 1 mL of culture media. Slices were kept in a CO_2_ incubator at 37°C, 5% CO_2_ for at least 2 days prior to use.

#### Animal preparation: acute experiments

Adult Sprague–Dawley rats of either sex (250–650 g) were anesthetized with 10% urethane (1.3 g/kg i.p.) and prepared for single unit recording as previously described (Turner et al. 2013). In brief, vascular access was obtained and rats were intubated and instrumented to record blood pressure, core temperature, and end‐tidal CO_2_. Rats were positioned in a stereotaxic frame in the skull flat or nose‐down (~30°) position. Bone overlying the brainstem was removed and the dura reflected. In experiments targeting respiratory neurons the caudal pole of the facial nucleus, an anatomical landmark for the respiratory cell column, was mapped by antidromic field potentials as previously described (Brown and Guyenet 1985). Diaphragmatic EMG was recorded as an index of respiratory phase via fine steel wire hook electrodes inserted through the thoracic wall into the diaphragm using a 26 gauge needle. When indicated by respiratory movements that interfered with recording stability, rats were artificially ventilated at parameters that maintained end‐tidal CO_2_ at 3.5–4.5% and movements suppressed by careful titration with pancuronium bromide (0.2–2 mg/kg i.v., AstraZeneca, North Ryde, NSW, Australia) such that diaphragmatic EMG was still observable. In long experiments, hydration and electrolyte balance was maintained by intravenous infusion of 0.9% NaCl or 5% glucose (5 mL/kg/h). Anesthetic depth was carefully monitored by examining autonomic, respiratory, and/or motor responses to firm pinch of the hindpaw; supplementary anesthesia (10% initial dose) was provided as required.

#### Animal preparation: recovery experiments

Adult Sprague–Dawley rats of either sex (85–605 g) were anesthetized with intraperitoneal ketamine (75 mg/kg; Parnell Laboratories, Alexandria, NSW, Australia) mixed with medetomidine (0.5 mg/kg; Pfizer Animal Health, West Ryde, NSW, Australia). Prophylactic antibiotics (20 mg/kg Cephazolin sodium, i.m.; Mayne Pharma, Salisbury South, SA, Australia) and analgesia (2.5 mg/kg Carprofen, s.c.; Norbrook Pharmaceuticals, Tullamarine, VIC, Australia) were administered and the left femoral artery and vein were cannulated under aseptic conditions. The brain was exposed as described above under minimally invasive conditions, medetomidine was reversed (atipamazole 1 mg; Pfizer Animal Health, Australia, s.c.), and anesthesia switched to 1–3% isoflurane (Veterinary Companies of Australia Pty Ltd, Artarmon, NSW, Australia) in 100% oxygen and monitored as described above for the remainder of the procedure. In some experiments rats were artificially ventilated following endotracheal intubation with a 14 gauge cannula.

After conclusion of recordings, wounds were irrigated and the exposed brain covered with oxidized cellulose hemostat. Neck muscles were sutured and skin closed with stainless steel suture clips. Femoral catheters were removed, vessels tied off, and incisions closed. Anesthesia was discontinued, rats were removed from the stereotaxic frame and, where applicable, extubated. Rats were treated with postoperative carprofen, (2.5 mg/kg s.c.; Norbrook Pharmaceuticals, Australia) and monitored closely for up to 36 h with additional analgesia as required.

### Histology

At the conclusion of in vivo experiments rats were euthanized with pentobarbitone (>100 mg/kg i.v. (acute experiments) or i.p.(recovery)), transcardially perfused with heparinised saline followed by 4% PFA, and the brain removed and postfixed in 4% PFA solution overnight. At the conclusion of in vitro experiments brain slices were briefly immersed in 4% PFA and transferred into TBPS until imaging.

Brainstems from in vivo experiments in which dextran or plasmids encoding fluorescent reporters were used were cut into 50 *μ*m coronal sections with a vibrating microtome, wet‐mounted, and immediately visualized under epi‐fluorescence. As part of a separate study, neurobiotin‐labeled neurons were processed for ChAT and somatostatin 2A receptor immunoreactivity before visualization. Sections were washed in 0.01 m phosphate buffered saline containing 0.2% Triton‐100 for 3 × 15 min, and incubated in 0.01 m phosphate buffered saline containing 2% bovine serum albumin and 0.2% Triton‐100 for 1 h at room temperature. Primary antibodies (Goat‐anti‐choline acetyltransferase, 1:800 (Chemicon, Millipore, Cat#AB144P), Rabbit anti‐SST 2a receptor 1:100 (Bio‐trend, Köln, Germany; ss‐8000‐rmc, Lot#a080826)), were added to the blocking buffer and sections were incubated for 48 h at 4°C. Sections were washed in TPBS 3 × 30 min and incubated in secondary antibodies (ExtrAvidin^®^‐FITC 1:500 (Sigma‐Aldrich, Sydney, NSW, Australia; Cat#E2760), Cy3^®^‐conjugated AffiniPure Donkey anti‐Goat IgG (H+L) 1:250 (Jackson ImmunoResearch Laboratories, INC, West Grove, PA; Code#705‐165‐147, Lot#68839), Alexa Fluor^®^ 647‐AffiniPure Donkey Anti‐Rabbit IgG (H+L) 1:250 (Jackson ImmunoResearch Laboratories, INC, Code#711‐605‐152, Lot#105115)) for 12 h at 4°C. Processed sections were washed again in TPBS 3 × 30 min before being mounted in serial order on glass slides and coverslipped for imaging with Zeiss Z1 (Carl Zeiss Pty Ltd, North Ryde, Australia) epifluorescent or Leica TCS SP5X (Leica Microsystems Pty Ltd, North Ryde, NSW, Australia) confocal microscopes. In two cases, CFP and EGFP immunoreactivity were enhanced using Rabbit‐anti‐GFP (1:1000, Life Technologies, Mulgrave, VIC, Australia; Cat# A‐6455).

### Recording parameters

Recordings were made using an Axoclamp 900A amplifier with HS‐9AX1 headstage (Molecular Devices, Sunnyvale, CA) in current clamp mode. This model has several features that make it suitable: its high maximum current output (1000 nA) is convenient for constant‐current electroporation, and the headstage is tolerant of externally generated voltages of up to 10 V.

Extracellular activity was simultaneously measured on two channels, one using conventional extracellular configuration (AC channel: Gain: 20–50, Band pass: 100–3000 Hz) and one configured for intracellular recordings (DC channel: Gain: 1, Band pass: DC‐3000 Hz). Data were sampled at 10 (AC) or 5 (DC) ksamples/s using a 1401plus or power1401 running Spike 2 version 7 (Cambridge Electronic Design, Cambridge, UK). AC recordings were played back as an audio signal during experiments.

Pipettes were pulled from filamented borosilicate glass (external diameter 1 mm, internal diameter 0.5 mm) using a P‐2000 pipette puller (Sutter Instruments, Novato, CA). Pipettes with a long taper and tip diameter of approximately 1 *μ*m (Resistance 10–20 MΩ when filled with 0.9% NaCl) were considered ideal for extracellular recordings. All pipettes were inspected using a microscope with a calibrated graticule.

Intracellular recordings were made using similar pipettes pulled to <1 *μ*m tip diameters and filled with either Tris EDTA buffer or water containing freshly filtered plasmid DNA diluted to a concentration of 250–350 ng/*μ*L in 1m KCl.

#### Constant voltage switching circuit

A constant voltage generator (DS2A‐mkII, Digitimer Ltd., Welwyn Garden City, Hertfordshire, UK) was connected in parallel to the recording pipette by connecting one pole of the stimulator to the pipette and the other to the experimental preparation. A high‐impedance recording circuit was maintained by isolating the stimulator from the pipette assembly with an electromagnet‐controlled reed switch. The increased capacitance (~7 pF) of the assembly was offset by the amplifier capacitance compensation. For electroporation the electromagnet was engaged with a 5 V TTL pulse and the stimulator triggered to produce the desired voltage train.

### In vitro electroporation

Experiments were performed on a patch electrophysiology rig under an Olympus microscope with differential interference contrast optics and immersion lenses. Pipettes were back loaded with plasmid DNA, fluorescent dextran (tetramethylrhodamine‐ or fluoroscein‐conjugated dextran (3000 MW, Invitrogen # D3307 and D3305, respectively, 1–3% in 0.9% NaCl), or neurobiotin (1–2% in 0.9% NaCl) and mounted on the recording headstage. Brain slices were placed in the recording chamber and perfused at 1–2 mL per minute. The pipette tip was guided onto the surface of the target cell until a dimple was formed. Pipette patency was maintained with positive pressure as required. Following electroporation the pipette was carefully retracted from the cell and reused until clogging occurred.

The same approach was used for transfection of neurons in organotypic culture, except pipettes were filled with freshly filtered plasmid DNA in 0.9% NaCl (0.3–3 *μ*g/*μ*L). SCE of organotypic cultures was typically completed within 15 min. Transfected neurons were washed in fresh media and restored to the incubator for 24–48 h before fixation and imaging of reporter‐expressing neurons.

### In vivo electroporation

Pipettes were prepared as above, mounted on the recording headstage and slowly (~10 *μ*m/s) lowered into the brainstem using a piezo microstepper. For extracellular recordings, pipette pressure was maintained at >200 mmHg until a the tip reached a depth of 2 mm at which point the pressure was reduced to 0–50 mmHg. The pipette was advanced in 3 *μ*m steps until a spontaneously active neuron was isolated and pressure was released. In many cases no pressure was applied beyond 2 mm deep, without any apparent effect on dye labeling efficiency or quality. Positive pressure was always used in attempted extracellular transfection.

Correct pipette position was verified by induction of an open‐cell response to single cell microstimulation. Once a recording from a single cell was isolated the pipette was withdrawn until spike amplitude was 0.2–0.5 mV and microstimulation was attempted. If no response was obtained the pipette was advanced and retested in 3 *μ*m steps until a response was observed (interpreted as establishment of contact) or the spike height receded (interpreted as passage of the pipette past the cell without making contact.

Electroporation was generally only attempted in neurons in which normal neuronal activity resumed after responses to microstimulation, although electroporation before recovery was still associated with robust labeling (see Fig. 5C). After electroporation the pipette was slowly withdrawn. Fresh pipettes were used for each track.

For the majority of intracellular recordings no pressure was applied during positioning of the pipette: it was lowered to a depth of 1.5 mm and then advanced in 1–5 *μ*m steps until an extracellular recording of a spontaneously active neuron was isolated. A capacitive buzz was applied to gain intracellular access and the pipette was further maneuvered until a stable membrane potential was obtained. After electrophoresis the pipette was slowly withdrawn in until the membrane potential returned to zero and was then withdrawn completely. New pipettes were used for each track.

### Plasmid preparation

Plasmids encoding fluorescent reporter proteins were used to validate electroporation: pCAG‐DsRed (Addgene: 11151), pCAG‐YFP (Addgene: 11180), pCAG‐EGFP (Addgene: 11150), pCAG‐CFP (Addgene: 11179), pCBA‐TdTomato (Addgene: 28017). Plasmids were amplified and purified according to the suppliers’ recommendations, filtered, and stored at −20°C until use.

### Avoidance of inadvertent neuronal labeling

Partial blockage of recording pipettes is commonly resolved by passing a high‐amplitude ‘clearing’ current through the pipette. In early experiments, we occasionally observed false‐positive dextran‐labeled neurons at locations at which anionic ‘clearing’ currents had been used; this can be avoided using clearing currents opposite in polarity to that of the dye. As was the case with in vitro experiments, in vivo electroporation sometimes labeled more than one neuron. This rarely happened with TMR‐dextran (2/79 recovered neurons, 2.5%), but occurred significantly more frequently with neurobiotin (7/51 neurons, 14%, *P *=**0.03, Fisher's exact test). Inadvertent labeling of neurons due to positive pressure ejection of dextran/neurobiotin was almost never seen; unintentionally labeled neurons were easily identified by their position dorsal to the end of the recording track.

## Results

### Dye‐labeling in vitro

**Figure 1.**
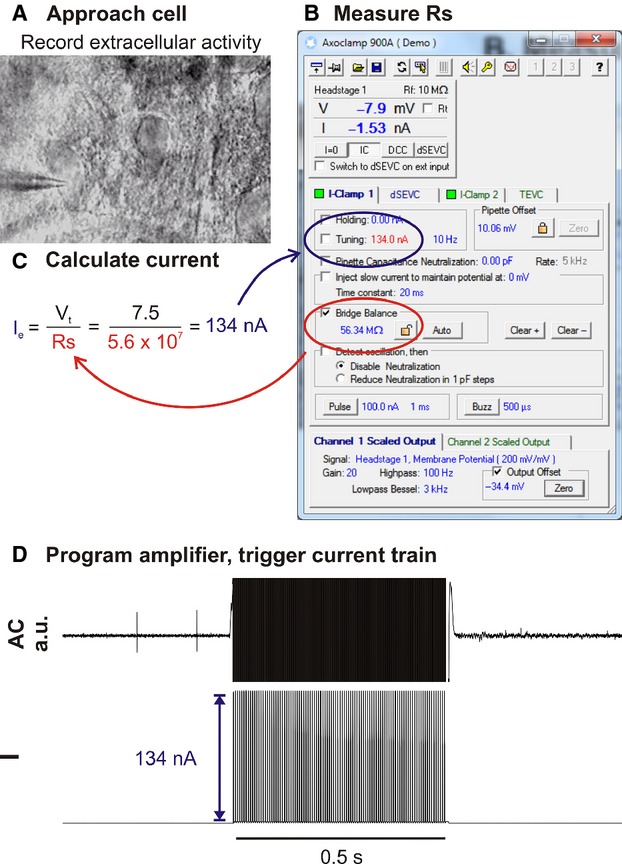
Illustration of workflow used for constant‐current electroporation in vitro. (A) Recording pipette is moved into gentle contact with the target cell under optical guidance. (B) Immediately prior to electroporation Rs is measured using the amplifier bridge‐balance function and (C) used to calculate the electroporation current (*I*_e_) required to generate the target voltage (*V*_t_; in this example 7.5 V). (D) The amplifier is programmed to deliver a train of current pulses at *I*_e_.

In pilot experiments performed on acute brain slices we first established that SCE was compatible with the micropipettes and recording amplifier used for extracellular recordings. Recording pipettes with a 1 *μ*m tip diameter were filled with 0.9% NaCl containing 1–3% fluorescein‐ or tetramethylrhodamine‐dextran (TMR‐dextran: MW 3000, resistance = 8–20 MΩ) and electroporation currents were delivered by the amplifier current ejection system. The pipette was positioned in gentle contact with the target cell under optical guidance and series resistance (Rs) was measured using the amplifier bridge‐balance function. The electroporation current (*I*_e_) required to generate the target electroporation voltage (*V*_t_) was calculated by Ohm's Law and programmed into the amplifier current‐injection dialogue (Fig. 1). As the amplitude of currents injected using this approach remain constant over the course of the electroporation train, we term this approach *constant‐current electroporation*.

**Figure 2.**
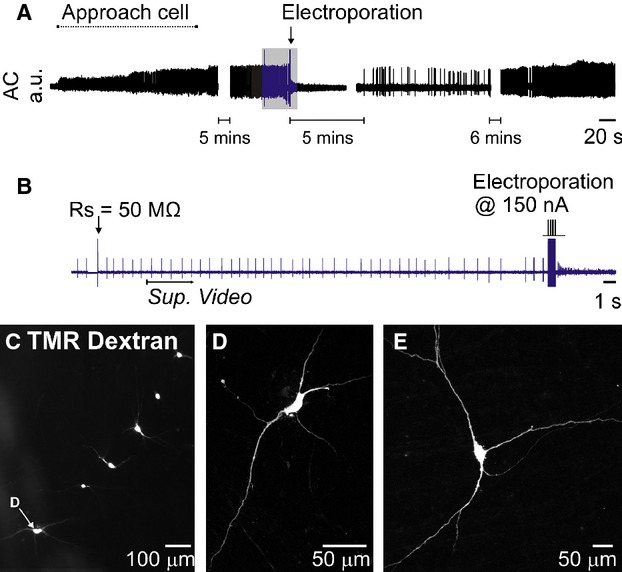
Extracellular recording and constant‐current electroporation of a spontaneously active neuron in an acute brain slice (same recording as Video S1). (A) Overview of entire recording. (B) Detailed view of portion indicated in blue in A. To electroporate Rs was first measured using the amplifier bridge‐balance function and used to calculate the appropriate electroporation current (see Fig. 1). A 200 Hz train of 100 × 150 nA, 1 ms pulses immediately filled the cell with 1% tetramethylrhodoamine (TMR)‐dextran and abruptly halted its spontaneous discharge. Spontaneous activity returned after five minutes and was maintained for the remainder of the experiment. *Sup. Video* indicates starting point of Supplementary Video 1. (C) Low‐power fluorescence image of six dextran‐filled neurons recorded and electroporated in a single slice (D) Confocal image of the neuron indicated in C. (E) Example of a neuron from a different experiment.

Constant‐current electroporation was compatible with high‐quality recording of extracellular action potentials and resulted in labeling of the soma and dendritic tree using a wide range of train parameters: reproducible single‐cell labeling was obtained with trains of 1 ms pulses delivered at 200 Hz for 0.5 s (*V*_t_ = 7.5 V: 36/48 cells labeled on the first attempt, Fig. 2, Video S1). Electroporation with even relatively low (<1%) concentrations of fluorescent dextrans resulted in extensive filling of fine axons and fibers, allowing resolution of fine morphological details (e.g. dendritic spines) that were clearly observable under epifluorescent illumination in the live slice. Resolution was enhanced by increasing the concentration of dextran used. In the vast majority of cases electroporation (successful or unsuccessful) caused an immediate cessation of spontaneous firing that rarely recovered within 10 min (although resumption of firing was not systematically investigated).

Electroporation occasionally labeled more than one cell (3/129 cells, 2.4%). In such cases the intended target was always labeled too, and processes extending from the unintentionally filled cell were always observed in close proximity to the recording pipette, and were therefore presumably labeled *en passant*.

### Single‐cell transfection in vitro

**Figure 3.**
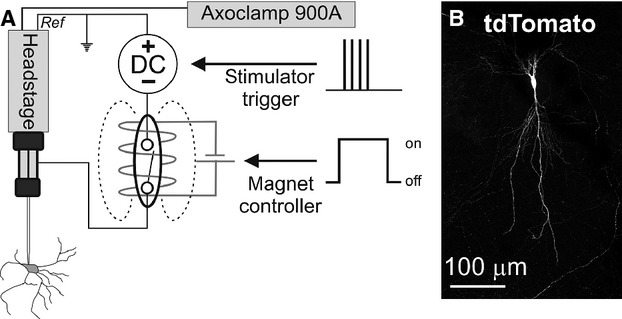
(A) Circuit configuration used for recording and constant‐voltage electroporation. A DC generator is connected in parallel to the recording and reference terminals of the amplifier headstage and is isolated from the recording electrode by a reed switch placed inside a cylindrical electromagnet. When the electromagnet is engaged the circuit is closed, allowing delivery of electroporation voltages. (B) Tdtomato fluorescence 24 h after transfection of a hippocampal neuron in organotypic culture with pCBA‐TdTomato.

Using the same approach we attempted to transfect neurons in organotypic cultures with plasmids that encode fluorescent proteins (*V*_t_ = −10 to −12 V, 100 × 0.5–1 ms pulses, 100 Hz, 1 s train). Although occasionally effective, this approach did not reliably result in protein transcription. As discussed in detail below, we postulated that low transfection efficiency may have resulted from voltage drop‐off during pore formation, so we modified our pipette assembly to incorporate a constant‐voltage source connected in parallel to the recording pipette by an electronic switch (“*constant‐voltage electroporation*”, Fig. 3A).

This modification allows delivery of constant‐voltage across the recording electrode (and headstage) when the switch is engaged, irrespective of Rs, but electrically isolates the voltage generator during when disengaged, preserving recording quality. This refinement improved the transfection efficiency achieved in organotypic cultures (reporter expression confirmed in 25/51 cells within 24 h of electroporation, Fig. 3B), making it equivalent to a commercial standalone SCE device (16/30 cells, Axoporator^™^, Fisher's Exact Test: *P *=**0.82) using the same electroporation parameters (−10 V, 100 Hz, 1 ms pulses, 1 s train). This modification also simplified the electroporation process, as it eliminated the requirement for measurement of Rs and calculation of *I*_e_ prior to electroporation. As described elsewhere, transfection of single neurons with plasmids that encode fluorescent reporters resulted in bright and complete filling of proximal and distal neuronal compartments, in most cases sufficient to clearly visualize fine branching of axons and dendrites, axonal varicosities and terminals, and dendritic spines (Haas et al. 2001).

### Establishing cell contact in blind recordings

SCE is critically dependent on gentle contact between the pipette and target neuron. In fields of view with a high cell density or recordings made deep in the slice it was often difficult to unambiguously determine when the recorded neuron had been correctly identified and contacted. Furthermore, in initial experiments in vivo*,* we were unable to achieve reliable electroporation when using changes in Rs to indicate cell contact (TMR‐dextran: 2/29 cells recovered, *n* = 4 rats), although other investigators have recently reported success using this strategy (Oyama et al. 2013).

**Figure 4.**
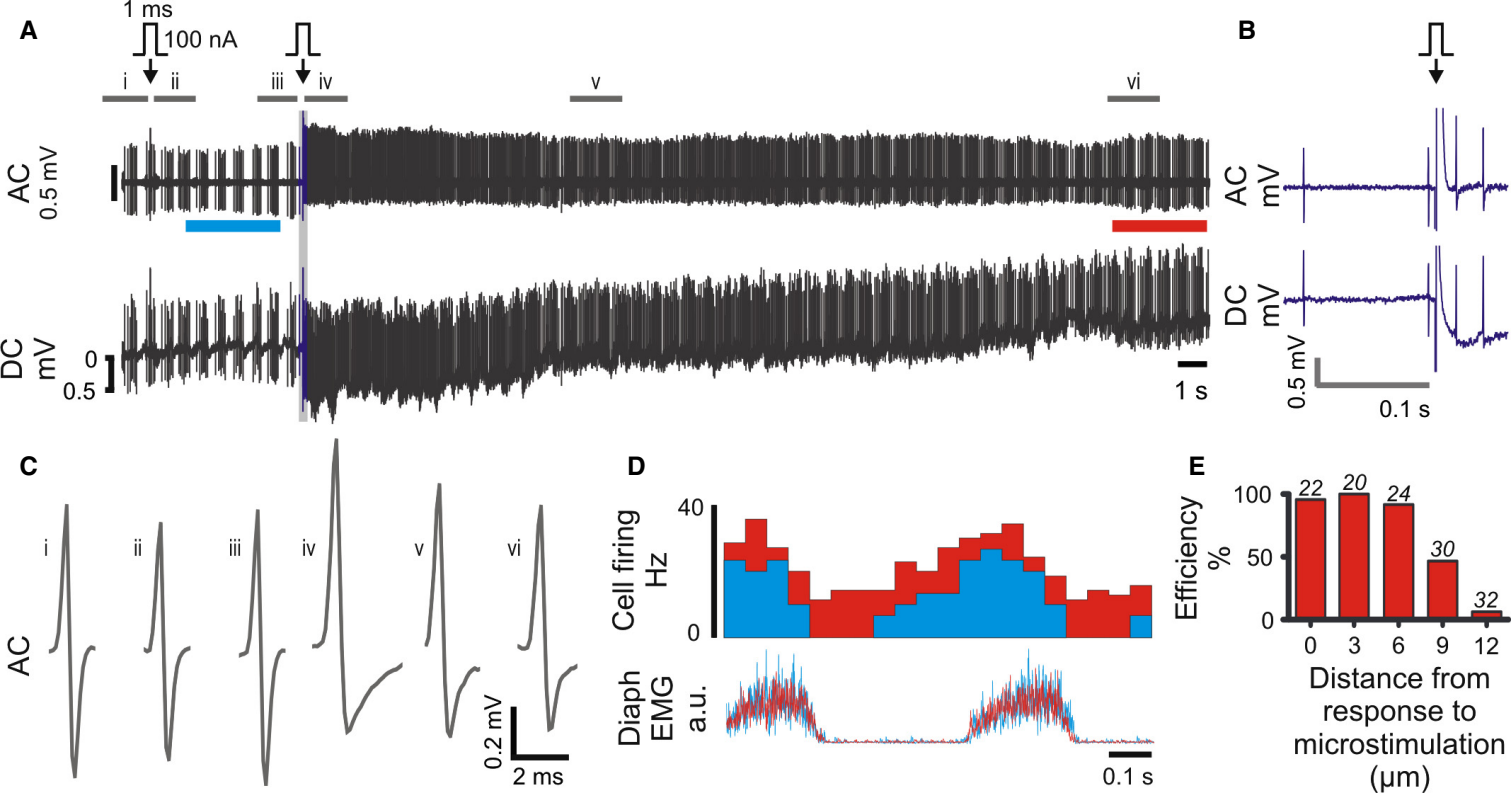
Single‐cell microstimulation of a medullary respiratory neuron in vivo. (A) 100 nA microstimulation (arrow) initially evoked no effect on neuronal firing. The pipette was advanced 3 *μ*m and stimulation repeated. This time the stimulus evoked transient stereotypical changes in firing frequency, spike amplitude and spike shape, apparent in the AC trace, and a small hyperpolarization of the pipette, visible as a 1 mV shift in the DC trace. (B) Expanded view of region drawn in blue in A. (C) Average spike waveforms; source data indicated in A. (D) Diaphragm‐triggered histograms of neuronal firing before (cyan, bar indicated in A) and after (red, bar indicated in A) microstimulation: the firing pattern is maintained over the recording. (E) Response to single‐cell microstimulation (0 *μ*m) is correlated with high labeling efficiency (TMR‐dextran, in vitro), which decreases as the pipette is withdrawn from the cell membrane. Number of replicates shown over each series.

To resolve this problem we developed a protocol that uses stereotypical responses to a single 50–100 nA, 1 ms pulse (“*single‐cell microstimulation*”) to definitively identify contact between the recording pipette and soma. In acutely prepared brain slices this stimulus evoked electrophysiological responses characterized by four distinct elements (Fig. 4): (1) Increased discharge rate and altered spike morphology that included (2) an increase in spike height and (3) adoption of an asymmetrical spike shape with a conspicuous after‐hyperpolarization (4) a 1–20 mV reduction in potential recorded in DC mode. All four components were *always* apparent in spontaneously active cells, but sometimes transient changes in pipette potential (with or without a short burst of action potentials) were the only features apparent in silent cells. Electrophysiological changes evoked by microstimulation typically recovered within 10 s and were never observed in the absence of physical contact between pipette and cell, confirmed as a dimpling of the cell membrane.

**Figure 5.**
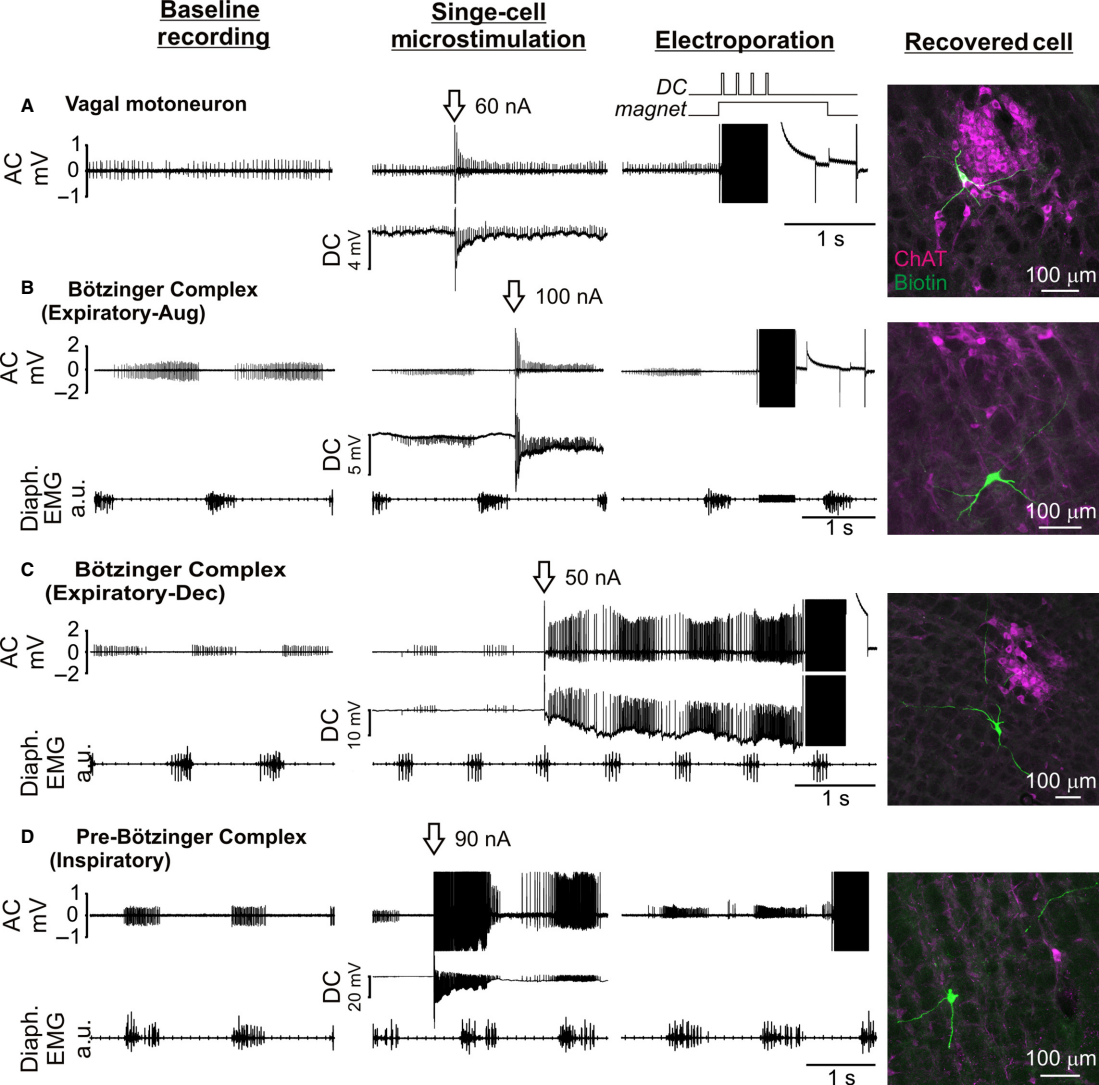
Examples of nonrespiratory (A) and respiratory (B–D) neurons recorded in extracellular mode in the ventrolateral medulla and labeled with neurobiotin by constant‐voltage electroporation in vivo. After establishment of baseline recordings and repositioning of the recording pipette such that spike height was <0.5 mV, single‐cell microstimulation (arrow) was used to verify cell contact. In most cases neurons were allowed to recover from microstimulation prior to electroporation (100 × 1 ms pulses, 200 Hz, +7.5 to +10 V). Photomicrographs show neurobiotin‐filled neurons (green) recovered at the appropriate stereotaxic co‐ordinates; magenta channel shows immunoreactivity for choline acetyl transferase (ChAT). Diaphragmatic EMG activity indicates inspiration, permitting functional identification of respiratory neurons. (A) Tonically active ChAT‐immunoreactive neuron in the nucleus ambiguus; (B) Augmenting expiratory neuron recorded ventral to nucleus ambiguus; (C) Decrementing expiratory neuron recorded ventral to nucleus ambiguus; (D) inspiratory‐locked neuron.

Although single‐cell microstimulation was apparently innocuous in vitro (where gentle contact between pipette and cell were closely monitored), microstimulation of spontaneously active neurons in vivo was initially associated with loss of recordings and presumed cell rupture and death. This was mitigated by withdrawal of the pipette from the cell once stable recordings were established such that spikes were <0.5 mV in amplitude prior to microstimulation. Where no response to microstimulation was observed the pipette was advanced in 1–3 *μ*m increments and stimulation repeated until a response was obtained. Using this approach transient responses, in which normal neuronal activity resumed within ~10 s, were achieved in approximately 50% of neurons (see Figs 4 and 5).

Responses to single‐cell microstimulation were a reliable indicator of contact between the pipette and cell and were predictive of successful electroporation. Under blind conditions in vitro*,* the probability of achieving dextran labeling on the first attempt was 89% (55/62 cells) following a positive response to single‐cell microstimulation, and rapidly declined as the pipette was withdrawn from the cell membrane (Fig. 4E). Positive responses to microstimulation were also correlated with successful constant‐current electroporation in randomly sampled spontaneously active neurons encountered 0.3–9.7 mm deep in vivo (dextran electroporation in 82/137 neurons, 60%, *n* = 26 rats, *V*_t_ = 5–10 V, 200 Hz, 1 ms pulses, 0.5 s train). A similar proportion (49/74, 66%, *P *=**0.37, Fisher's exact test) were labeled using dextran or neurobiotin in a second cohort of experiments (*n* = 21 rats) in which neurons in the ventrolateral medulla with respiratory‐related activity were preferentially targeted with constant‐voltage electroporation (7.5–10 V, 200 Hz, 1 ms pulses, 0.5 s train: Fig. 5).

### Single‐cell transfection in vivo

Having verified that constant‐voltage electroporation is capable of reliable single‐cell transfection in vitro and established a protocol that results in reproducible dye‐labeling in vivo, we then examined its suitability for single‐cell transfection in vivo.

Brainstem neurons were recorded 1.6–9.8 mm deep in either nonrecovery experiments, in which urethane anesthesia was maintained for 12–18 h after electroporation (*n* = 5 rats), or recovery experiments (*n* = 7 rats), in which anesthesia was reversed at the conclusion of recording and rats were recovered for 1–2 days. Contact between the pipette and target neuron was first verified by observing a positive response to single‐cell microstimulation, and neurons that recovered were electroporated at negative polarity (−10 V, 50–100 Hz, 0.5–1 ms pulses, 1 s). Regardless of surgical preparation, electroporation parameters, or plasmid construct used, this approach rarely resulted in reporter expression (6/87 neurons, 7%).

We initially hypothesized that the low success rate may have reflected an incorrect assumption regarding the predictive value of our microstimulation technique. We adopted an approach similar to that used for electroporation of superficial cortical neurons (Judkewitz et al. 2009; Oyama et al. 2013), in which the pipette is maneuvered such that Rs is increased by 30%, and attempted SCE in 31 neurons in five recovery experiments; no transfected cells were subsequently identified.

**Figure 6.**
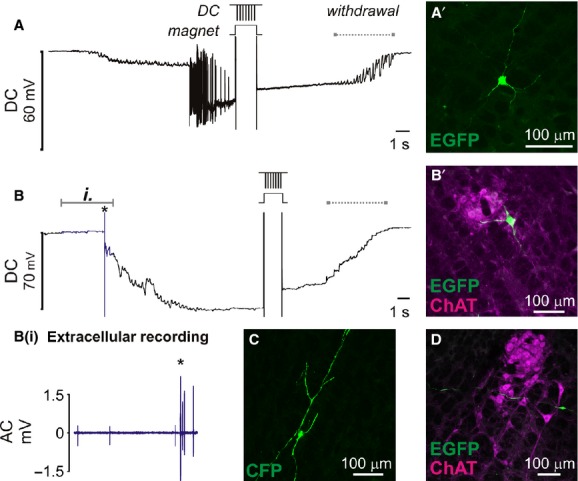
Transfection of ventrolateral brainstem neurons following intracellular penetration in vivo. Once membrane potential had stabilized plasmid DNA encoding fluorescent protein was electrophoretically injected by −10 V pulses (50 × 1 ms pulses, 1 s). Membrane potential was retained after electrophoresis until withdrawal of the pipette (indicated by dashed lines). (A) Electrophysiological recording from a silent neuron that started firing after penetration. A’ shows EGFP‐labeled neuron recovered at the corresponding stereotaxic coordinates. (B) Slowly firing spontaneously active neuron: extracellular spikes (blue data, detailed in Bi.) were resolved prior to cell penetration (*). (B’) Colocalization of EGFP with ChAT immunoreactivity indicates that this example is a cholinergic motor neuron in nucleus ambiguus. (C + D) show examples of other neurons transfected using the same approach.

Review of recordings from successfully transfected neurons revealed that microstimulation had in some cases resulted in full intracellular access prior to electroporation (5/6 neurons), suggesting that transfection could be achieved by direct intracellular plasmid electrophoresis. This hypothesis was examined in experiments in which brainstem neurons were targeted for intracellular recordings using semisharp pipettes (tip diameter: <1 *μ*m, resistance 18 ± 3 MΩ). This configuration was compatible with stable recording of unit activity in extracellular mode prior to brief (typically <10 s) intracellular access and plasmid ejection (−10 V, 50 Hz, 1 ms pulses, 1 s train or −10 V, 1000 Hz, 0.1 ms pulses, 0.1 s train: Steinmeyer and Yanik (2012)). Transfection was achieved in 13/48 (27%) neurons in which intracellular access (membrane potential −30 to −70 mV) was obtained prior to electrophoresis (Fig. 6). Transfected neurons were recorded 1.6–8.3 mm deep to the brain surface using pipettes between 8 and 37 MΩ in resistance. 3/13 neurons were silent; the remainder showed spontaneous activity between 1 and 20 Hz. Cross‐sectional areas of transfected neurons were 141–1178 *μ*m^2^, equivalent to 13–39 *μ*m in diameter.

## Discussion

The current study provides researchers with three novel protocols that can be used for reliable dye‐labeling or transfection of functionally identified neurons in deep brain regions in vivo. Constant‐current electroporation can be performed without any customization of the recording amplifier and yields rapid and high quality dye labeling in vitro and in vivo. Constant‐voltage electroporation requires minimal modification of hardware and yields in vitro transfection at efficiency equivalent to a widely used proprietary device, but in our hands this approach was incompatible with high transfection efficiency in vivo. In contrast, we found that intracellular electrophoresis of genetic material may instead offer a more reliable method for transfection of functional identified neurons in inaccessible brain regions.

In establishing our transfection methodology we developed dye‐labeling techniques that offer some advantages to the juxtacellular method in terms of simplicity and speed. Successful juxtacellular labeling is indicated by ‘entrainment’ of neuronal firing to regular anodal pulsing of the recording pipette, which must be maintained for several minutes for reasonable labeling to occur. Longer periods of entrainment are associated with more complete labeling (up to 60 min: Noseda et al. 2010). Although the juxtacellular approach can yield efficient labeling in the hands of the technically elite (100/115 cells: Pinault 1996; 45/50 cells: Guyenet and Wang 2001), establishing and maintaining entrainment requires considerable finesse, and recordings are often lost before or during entrainment. It is notable that the number of neurons lost during attempted labeling is often omitted from reports that use this approach, perhaps boosting the apparent efficiency of the technique: in unpublished pilot experiments we found that recordings were lost before or during entrainment in 145/248 (58%) attempts.

In contrast with the imprecise cues used to guide entrainment over minutes of juxtacellular labeling, single‐cell microstimulation provides an instant binary output; a cell either responds with an unambiguous electrophysiological signature, in which case it may be immediately electroporated with a high probability of recovery, or does not, in which case the pipette is maneuvered and stimulation repeated (in cases where there is no response) or another cell is sought (in cases where the recording is lost). This makes the protocol quick to perform and easy to learn: most of the experiments targeting respiratory neurons were performed by a graduate student (SL) who learned the technique in a week and recovered two labeled neurons in his first experiment.

The increase in neuronal excitability and effects on spike shape and amplitude seen in response to microstimulation are consistent with induction of an “open‐cell” state by the stimulus, a transient permeabilization of the neuronal membrane evoked by its partial electroporation (Braeken et al. 2012; Spira and Hai 2013). Our observation that such responses only occur when the pipette is in contact with the neuron are supported by similar reports by Santos et al. (2007, 2009), who found that 30–100 nA, 1 ms pulses could be used to effectively stimulate spinal cord neurons in loose‐seal mode, but not in the absence of physical contact between the pipette and cell. We conclude that single‐cell microstimulation provides a reliable and objective indicator of contact between the pipette and neuron. The major shortcoming associated with using single‐cell microstimulation to guide electroporation is its high attrition rate in vivo; this is most likely due to excessive contact between the pipette and target cell, as it does not seem to occur in vitro (where contact can be closely monitored), although factors such as cell size and pipette geometry may also contribute to the effect.

Constant‐current and constant‐voltage electroporation offer dye‐labeling with similar efficiencies in vitro and in vivo; both resulting in labeling that is at least equivalent in quality to that offered by the juxtacellular technique in vivo and comparable to standard SCE in vitro, where fine morphological features such as dendritic spines and terminals are consistently revealed (Haas et al. 2001; Umeda et al. 2005). The quality of labeling obtained using the juxtacellular technique varies according to the brain region targeted and tissue processing, hampering direct comparison with the protocols described here. However, extensive filling of brainstem neurons juxtacellularly labeled with neurobiotin and visualized with fluorescent avidin conjugates is rare in the literature (Sartor and Verberne 2003; Abbott et al. 2009; Kanbar et al. 2011; Boucetta et al. 2014; Iceman and Harris 2014) and in our own experience (unpublished data). In contrast, although variability in labeling quality was observed using the approaches described here, we often saw extensively filled neurons that projected across dozens of histological sections in which fine morphological details were visible. We did not extensively investigate the quality of labeling possible using diaminobenzidine visualization, but details such as terminals and dendritic spines were visible in one of three neurons processed that way. Constant‐current electroporation may be conducted without modification to hardware provided the recording amplifier is capable of generating sufficiently high currents (400–800 nA), making it fast and cheap to adopt. However, we found it more convenient to use the constant‐voltage approach, as it eliminates measurement of Rs and current calculation from the workflow, speeding up the protocol, and is compatible with single‐cell transfection in vitro.

Why does not constant‐current electroporation result in transfection? In constant‐current mode the measured value of Rs has a critical influence on voltage output. Constant‐current pulses may initially succeed in generating V_t_ for a given Rs; however, pores are formed in the membrane within microseconds of voltage application (see DeBruin and Krassowska 1999; Wang et al. 2010), lowering membrane resistance and consequently Rs, resulting in a proportionate reduction in trans‐membrane voltage. As a consequence of this voltage drop‐off, constant‐current electroporation may fail to sustain the voltages required for large and stable pore formation, which are crucial for efficient plasmid delivery (Rae and Levis 2002).

In vitro transfection efficiency was restored by integration of a constant‐voltage generator to the recording circuit. This modification allowed the delivery of up to ±10 V without risk to the recording headstage (following advice from the manufacturer), and made transfection efficiency equivalent to that obtained with a commercial single‐cell electroporator and comparable to that reported elsewhere (Rae and Levis 2002; Rathenberg et al. 2003; Steinmeyer and Yanik 2012). However, exhaustive attempts to translate it for single‐cell gene delivery in vivo were fruitless. This is surprising, given the similarities between our approach and protocols recently described by other investigators, in which some level of transfection was observed under all parameters tested (Cohen et al. 2013; Oyama et al. 2013). Differences in the brain regions and, perhaps crucially, the depths at which neurons were targeted, may underlie this disparity, as Cohen et al. (2013) and Oyama et al. (2013) restricted their attempts to neurons within 450 *μ*m or 1.5 mm of the brain surface, respectively, where pipette patency is easier to manage.

We conclude that techniques based on SCE are unlikely to yield reliable transfection of neurons in deep brain structures, and speculate that blockage of pipettes is most likely responsible for the poor transfection efficiency seen in vivo. Despite applying high positive pressure to the internal solution during brain penetration and cell hunting, we rarely saw any evidence of dye leakage or hydraulic injury along pipette tracks, both of which indicate pipette patency in vivo (Rancz et al. 2011), and the quality and efficiency of dye‐labeling and reporter expression were consistently higher in vitro than in vivo. Although we were able to use SCE for efficient dye‐labeling nearly 10 mm deep to the brain surface, this does not necessarily mean that recording pipettes were patent: in our experience electrophoretic ejection of fluorescent dextran is consistently possible from clogged pipettes in which no dextran may be pressure‐ejected, and we were able to produce robust labeling with such pipettes in vitro. However, we and others have found that blocked pipettes absolutely preclude transfection by SCE (Haas et al. 2001; Rae and Levis 2002; Rathenberg et al. 2003; Bestman et al. 2006; Kitamura et al. 2008; Judkewitz et al. 2009).

If patency is the main issue affecting the efficiency of SCE in deep brain regions, pipette clogging probably reduces rather than completely obstructing plasmid ejection, as intracellular plasmid electrophoresis reproducibly transfected neurons up to 8.3 mm deep. Stable intracellular access is difficult to achieve even in acute preparations, where extensive craniotomy or pneumothoraces are commonly used to reduce movement. However, we found that brief access, sufficient for transfection, could be gained in minimally invasive preparations (although we found tracheal intubation with neuromuscular block and artificial ventilation useful) and that large neurons could be impaled and transfected using low‐resistance pipettes. The current data provide a proof‐of‐principle that intracellular recording may be used to transfect neurons in deep brain regions, but the efficiency of the approach will ultimately dependend on factors including operator experience, animal age, brain region targeted, and the size of targeted neurons.

The current data provide relatively simple protocols that can be used for reliable and robust labeling of recorded neurons and a novel approach for transfection of neurons in deep brain regions. The clear‐cut criteria used to guide electroporation and the rapidity at which labeling can be performed may prove particularly attractive to novice investigators, whereas the potential to select neurons for genetic modification based on their functional properties may prove useful to investigators interested in applying advanced connectome‐tracing technologies at single‐cell resolution (Wickersham et al. 2007; Marshel et al. 2010; Rancz et al. 2011).

## Conflict of Interest

None declared.

## Supplementary Material

Supplementary Video S1
